# Factors of suicide-related behaviors based on stress-vulnerability model and prevention strategies among nurses: a scoping review

**DOI:** 10.3389/fpsyg.2025.1483904

**Published:** 2025-01-20

**Authors:** Xiaoyu Yang, Deying Hu, Lecheng Li, Rezvanguli Rezak

**Affiliations:** ^1^School of Nursing, Tongji Medical College, Huazhong University of Science and Technology, Wuhan, China; ^2^Department of Nursing, Union Hospital, Tongji Medical College, Huazhong University of Science and Technology, Wuhan, China

**Keywords:** nurse, suicide, current situations, the stress-vulnerability model, tertiary prevention

## Abstract

**Objective:**

The objective of this scoping review was to explore, appraise and synthesize the current literature regarding the incidence, factors influencing, and prevention strategies related to suicide risk among nurses.

**Methods:**

An extensive literature search was conducted using databases such as PubMed, Web of Science, Medline, and Embase from its formation to June 20, 2024, specifically focusing on the suicide-related behaviors of nurses written in Chinese or English. Two researchers independently screened the literature, and disagreements were debated until a consensus was reached. Data extraction was conducted for the studies that were included. The process of data synthesis was carried out using narrative analysis.

**Results:**

The study encompassed 40 papers from 15 different countries. This study found that nurses’ suicide ideation ranged from 4.3 to 44.58%, while suicide attempts ranged from 2.9 to 12.6%. Based on the stress-vulnerability model, factors influencing nurses’ suicide-related behaviors include vulnerability (personality traits, coping styles), stressors (mental disorders, workplace bullying, etc.) and protective factors (social support, resilience, etc.). The strategies for preventing nurse suicide encompass primary prevention (for all nurses), secondary prevention (for nurses at risk of suicide), and tertiary prevention (for nurses who have attempted suicide).

**Conclusion:**

The suicide rate among nurses exceeds that of the general population. Mental disorders and workplace bullying are significant stressors that contribute to nurse suicide. Suicide-related behaviors among nurses can be effectively prevented and managed through the implementation of the tertiary prevention strategies. Primary prevention is essential in reducing suicide. Cognitive exercises and schedule shifts reasonably are primary preventive measures tailored for nurses. This study addresses the gaps in influencing factors about suicide-related behaviors among nurses and the strategies for preventing suicide, and provides a complete review of the current situation of nurses’ suicide-related behaviors, providing references for the safe management of nurses’ suicide.

## 1 Introduction

Suicide is an enduring global public health issue that is of great concern, causing severe and enduring detrimental impacts on society at large. Based on the most recent data provided by the World Health Organization, the global suicide rate stands at over 700,000 deaths annually, which represents 1.3 percent of all fatalities (World Health Organization, 2019). Suicide-related behavior constitutes a psychiatric emergency that poses a significant threat to an individual’s life. This includes non-suicidal self-injury (NSSI), suicidal ideation (SI), suicide attempt (SA), and completed suicide (CS) (Fen et al., 2019). Non-suicidal self-injury can be differentiated from other suicidal behaviors based on the absence of a wish to die. Studies have shown that non-suicidal self-injury (NSSI) increases the risk of suicide by 40 times and is the most significant predictive risk factor for suicide (Hamza et al., 2012) Suicidal ideation is differentiated from suicide attempt and completed suicide based on whether or not an individual engages in a suicidal act. The primary distinction between a suicide attempt and a completed suicide lies in whether the act ultimately led to a deadly outcome (Fen et al., 2019).

Nurses constitute a unique professional classification. Nursing job is fraught with numerous stressors, including challenging working conditions and recurrent physical and psychological aggression, rendering nurses a high-risk category for suicide. Nevertheless, the majority of research on suicide has focused on suicidal behaviors in students, individuals with mental problems, and the general population, resulting in a scarcity of studies addressing suicidal behaviors among nurses. An American cohort study revealed a greater incidence of suicide among nurses compared to the general populace (Davis et al., 2021). The suicide-related behaviors of nurses not only cause significant psychological distress to those around them but also directly impact the quality of healthcare services. Paying attention to nurses’ suicide-related behaviors and their influencing factors, reducing the incidence and mortality of suicide-related behaviors, is of great practical significance for the stability of the healthcare team and the sustainable development of human healthcare.

As for the mechanism of suicide-related behaviors, the more recognized explanation in the academic community is the “stress-vulnerability” model theory proposed by Mann et al. In general, the “stress-vulnerability” model is considered to be the most pathological and explanatory model for suicidal behavior under the pathogenic model of mental illness (Mann et al., 1999). The model has been applied to a variety of populations, such as prison populations, veterans, and college students, but has not been applied to nursing populations (Bonner and Rich, 1988; Rozanov and Carli, 2012; Scott et al., 2023). Applying the stress–vulnerability model encourages a more sophisticated formulation, which considers not only the vulnerabilities and coping skills of the nurses but also static and dynamic factors related to the hospital environment. Understanding the factors that contribute to suicide-related behaviors in this population and implementing targeted preventive interventions early on can help prevent and reduce suicide-related behaviors. Currently, multifactorial and complicated diseases are handled by tertiary prevention. According to this, researchers have suggested a tertiary prevention of suicide, which is currently widely employed for patients, college students, and adolescents.

Therefore, this study will explore the influencing factors and prevention strategies of nurse suicide based on the stress-vulnerability model and the tertiary prevention model of suicide, providing a reference for the safe management of nurse suicide.

## 2 Materials and methods

In seeking an answer to the overarching question, a scoping review was conducted to provide an overview of the literature on suicide-related behaviors among nurses (Sucharew and Macaluso, 2019). A scoping review is a crucial step in assessing the potential dimensions and scope of existing research literature, with the aim of determining the nature and extent of the research evidence (Peters et al., 2020). This methodology was well-suited to the objectives of our study, as our goal was to understand the current status of suicide-related behaviors among nurses, the factors influencing these behaviors, and effective prevention strategies. From the outset, we established the search strategy and exclusion criteria in accordance with the methodology, which guided us in defining the scope of the paper. Additionally, we conducted a thorough quality assessment of the obtained articles, which is elaborated upon in this section. The final step involved synthesizing and reporting the results.

### 2.1 Search strategy

A computerized search was performed to obtain publically published research material on suicide-related behaviors among nurses from the PubMed, Web of Science, Medline, and Embase databases until June 20, 2024. The search terms were used in a combination of subject and free word searches, with the following keywords: (suicid* OR self-injur* OR self-harm OR self-destructive) AND (Nurse* OR Nursing Personnel).

### 2.2 Eligibility criteria

Inclusion Criteria: The study types included observational studies or experimental studies; Participants must be registered nurses. The outcome measures were the incidence and/or influencing factors of suicide-related behaviors and/or strategies for prevention. Exclusion Criteria: Case reports, reviews, duplicate publications, and dissertations; studies from which incident-related data could not be extracted or translated; studies of substandard quality; texts that are entirely inaccessible; and literature not published in Chinese or English.

### 2.3 Study selection and data extraction

**Figure 1:**
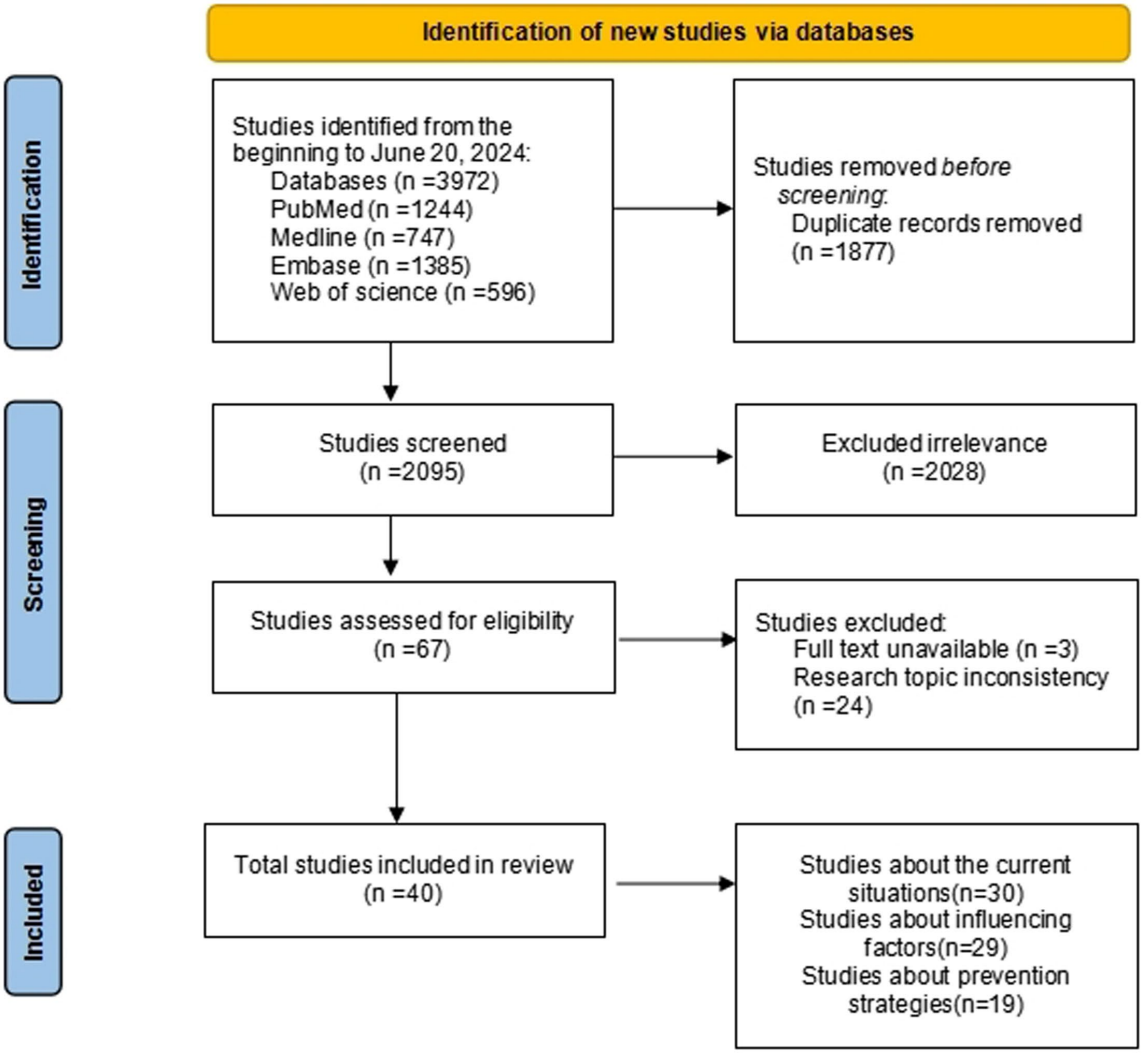
Flow chart for literature search.

Two researchers proficient in evidence-based nursing systems did the literature screening independently, where they examined the titles and abstracts of publications and initially excluded those that were not significantly relevant. Following the initial screening, the literature was thoroughly examined for secondary screening and subsequently cross-referenced for compilation. When inconsistencies occurred, both sides engaged in discussions and negotiations to achieve a consensus, and sought a third-party perspective if needed. The literature search flowchart is displayed in Figure 1.

### 2.4 Quality of assessment

Cohort studies, longitudinal studies, and case–control studies were evaluated using the Newcastle-Ottawa Scale (Stang, 2010), an 8-item scale with a maximum score of 9. Scores ranging from 0 to 4 indicate low quality, 5–6 indicate moderate quality, and 7–9 indicate high quality. Cross-sectional studies were assessed using the Agency for Healthcare Research and Quality (AHRQ) evaluation tool. The AHRQ standard consists of 11 items, with a total possible score of 11 points (Pede and Uguccioni, 2001). A total score of 0–3 is classified as low quality, 4–7 as moderate quality, and 8–11 as high quality.

## 3 Current situation of suicide-related behaviors among nurses

There have been about 30 studies that have investigated the incidence of nurses’ suicide-related behaviors in 14 different countries. There is not enough research on suicide among nurses in comparison to other populations, with a particular emphasis on studying suicidal ideation among nurses. Table 1 presents a summary of the information about suicide-related behaviors among nurses.

**Table 1:** Literature information summary.

Author(s)	Country	Methods	Population	Time	Sample size	Results	Quality assessment (points)
Merkle et al. (2023)	USA	Cross-sectional study	School nurses	2022	7,971	4.3 percent had suicidal ideation.	7
Kelsey et al. (2021)	USA	Cross-sectional study	Hospital nurses	2017	86,858	5.5 percent had suicidal ideation in the past year.	7
Davis et al. (2021)	USA	Retrospective cohort study	Nurses	2007–2018	159,372	From 2007 to 2018, 2,374 nurses committed suicide. The 2017 suicide rate for female nurses was 23.8 per 100,000 person-years, compared to 31.1 for male nurses.	6
Patrician et al. (2020)	USA	Case–control study	Nurses	2015	NA	Suicide rate for female nurses is 11.4 per 100,000 person-years, compared to 29.3 per 100,000 person-years for male nurses.	6
Davidson et al. (2019)	USA	Case–control study	Nurses	2014	NA	Suicide rate for female nurses is 11.97 per 100,000 person-years, compared to 39.8 per 100,000 person-years for male nurses.	6
Davidson et al. (2018)	USA	Case–control study	Nurses	2005–2015	NA	Suicide rate of 18.51 per 100,000 person-years.	7
Feskanich et al. (2002)	USA	Retrospective cohort study	Nurses	1982–2002	94,110	Suicide rate 6.8 per 100,000 person-years.	
Wang et al. (2023)	China	Cross-sectional study	Hospital nurses	2021	787	7 percent had suicidal ideation, 3 percent had suicidal plan.	6
Lu et al. (2023)	China	Cross-sectional study	Hospital nurses	2018	1901	16.8 percent had suicidal ideation, 10.8 percent had attempted suicide in the past year.	6
Peng et al. (2022)	China	Cross-sectional study	Hospital nurses	2020	6,183	22 percent had suicidal ideation.	6
Gonzalez Mendez et al. (2022)	China	Cross-sectional study	Hospital nurses	2020	573	9.9 percent had suicidal ideation.	5
Xu et al. (2021)	China	Cross-sectional study	Hospital nurses	2020	1,507	6.47 percent had suicidal ideation.	6
Hong et al. (2021)	China	Cross-sectional study	Hospital nurses	2020	4,692	6.5 percent had suicidal ideation.	7
Wang et al. (2020)	China	Cross-sectional study	Hospital nurses	2018	1,531	10.8 percent had suicidal ideation.	6
Chin et al. (2019)	China	Cross-sectional study	Hospital nurses	2014	2,734	18.3 percent had suicidal ideation.	6
Cheung and Yip (2016)	China	Cross-sectional study	Hospital nurses	2013	850	14.9 percent had suicidal ideation and 2.9 percent had attempted suicide.	5
Cheung et al. (2016)	China	Cross-sectional study	Hospital nurses	2013	850	9.3 percent self-injury in the past year.	6
Zhou et al. (2004)	China	Cross-sectional study	Hospital nurses	2004	470	5.3 percent had suicidal ideation.	5
Kantorski et al. (2022)	Brazil	Cross-sectional study	hospital nurses	2020	890	7.4 percent had suicidal ideation.	6
Freire et al. (2020)	Brazil	Cross-sectional study	Hospital nurses	2018	216	9.94 percent had suicidal attempts.	6
Brady et al. (2023)	Ireland	Cross-sectional study	Hospital nurses	2021	166	11 percent had suicidal ideation and 4 percent had suicide plan.	6
Brady et al. (2022)	Ireland	Cross-sectional study	Nursing homes nurses	2020	390	25 percent had suicidal ideation and 16 percent had suicide plan.	6
Groves et al. (2024)	England	Case–control study	Nurses	2010–2020	NA	Self-injurious behavior occurred in 81 nurses and was repeated in 17.3 percent of nurses.	6
Dalum et al. (2024)	Norway	Retrospective cohort study	Nurses	1980–2021	133.6 million	Suicide rate 22.1 per 100,000 person-years.	
Alyahya et al. (2023)	Saudi	Cross-sectional study	Hospital nurses	2021	419	24.58 percent had suicidal ideation.	5
Badrfam et al. (2023)	Iran	Cross-sectional study	Hospital nurses	2020	305	22 percent had suicidal ideation.	6
Kabir et al. (2023)	Bangladesh	Cross-sectional study	Hospital nurses	2021	1,264	13.26 percent had suicidal ideation.	6
Martinez-Arriaga et al. (2023)	Mexico	Cross-sectional study	Hospital nurses	2021	158	1.2 percent had attempted suicide in past three months.	5
Höller and Forkmann (2022)	German	Cross-sectional study	Hospital nurses	2021	1,311	21.7 percent had suicidal ideation in the past month, and 44.5 percent had suicidal ideation. 0.5 percent of nurses had attempted suicide in the past month and 12.6 percent had attempted suicide.	6
Stelnicki et al. (2020)	Canada	Cross-sectional study	Hospital nurses	2020	3,969	Suicidal ideation (10.5 percent, 33.0 percent), plans (4.6 percent, 17.0 percent) and attempts (0.7 percent, 8.0 percent) in the past year and/or lifetime.	7

There is not enough research on non-suicidal self-injury among nurses. A survey conducted in Hong Kong found that 9.3% of nurses have participated in self-harming behavior in the past year (Cheung and Yip, 2016). Another study in the United Kingdom reported that 17.3 percent of nurses who took place in self-injurious behaviors would repeat the behavior (Groves et al., 2024). There is a significant amount of research on the rate of suicidal ideations among nurses. The lifetime incidence rates vary between 4.3 and 44.58% (Alyahya et al., 2023; Badrfam et al., 2023; Brady et al., 2022; Brady et al., 2023; Cheung et al., 2016; Chin et al., 2019; Gonzalez Mendez et al., 2022; Höller and Forkmann, 2022; Hong et al., 2021; Kabir et al., 2023; Kantorski et al., 2022; Kelsey et al., 2021; Liu et al., 2023; Lu et al., 2023; Merkle et al., 2023; Peng et al., 2022; Stelnicki et al., 2020; Wang et al., 2020; Xu et al., 2021; Zhou et al., 2004). There is a notable variation in the incidence of suicidal ideation among nurses across different countries. In China, the rate ranges from 5.3 percent to 22 percent, while in the US, it is between 4.3 percent and 5.5 percent. Further studies also showed that nurses in different work environments had varying rates of suicidal ideation. School nurses were less likely to do so than hospital nurses, although nursing home nurses were more likely to do so. A study that examined the prevalence of suicidal ideation among school nurses discovered that 4.3% of them experienced it (Kabir et al., 2023). As a result of Brady’s research, more nursing home nurses had suicidal ideation and plans than hospital nurses (Brady et al., 2022; Brady et al., 2023). Furthermore, four additional studies found that nurses had more serious suicidal ideation (i.e., suicidal plan), with rates ranging from 3 to 17 percent (Brady et al., 2022; Brady et al., 2023; Stelnicki et al., 2020; Wang et al., 2023).

Research investigating suicide attempts among nurses revealed that the prevalence of such attempts varied between 2.9 and 12.6%. Within the last month and 3 months, 0.5 and 1.2% of nurses attempted suicide, respectively. Over the last year, the percentage of nurses who attempted suicide went from 0.7 to 10.8%.(Cheung et al., 2016; Freire et al., 2020; Höller and Forkmann, 2022; Lu et al., 2023; Martinez-Arriaga et al., 2023; Stelnicki et al., 2020). Only two nations conducted research on the mortality rate of nurses due to suicide. The suicide death rate for nurses in Norway between 1980 and 2021 was 22.1 per 100,000 person-years (Dalum et al., 2024). From 2005 to 2015, the suicide rate in the United States was 18.51 per 100,000 person-years, significantly surpassing the nursing suicide fatality rate of 6.8 per 100,000 person-years in 2002 (Davidson et al., 2019; Davidson et al., 2018; Davis et al., 2021; Feskanich et al., 2002; Patrician et al., 2020).

The variability in the research data can be mainly attributed to (1) the substantial disparity in the number of patient samples. (2) Survey participants differ in age, marital status, geography, and nursing specialty, affecting study results. (3) Varying national circumstances and cultural norms throughout different countries. (4) The time frame is limited and can vary.

## 4 Influences on nurses’ suicide-related behaviors based on the stress-vulnerability model

### 4.1 An overview of the model of stress-vulnerability

**Figure 2:**
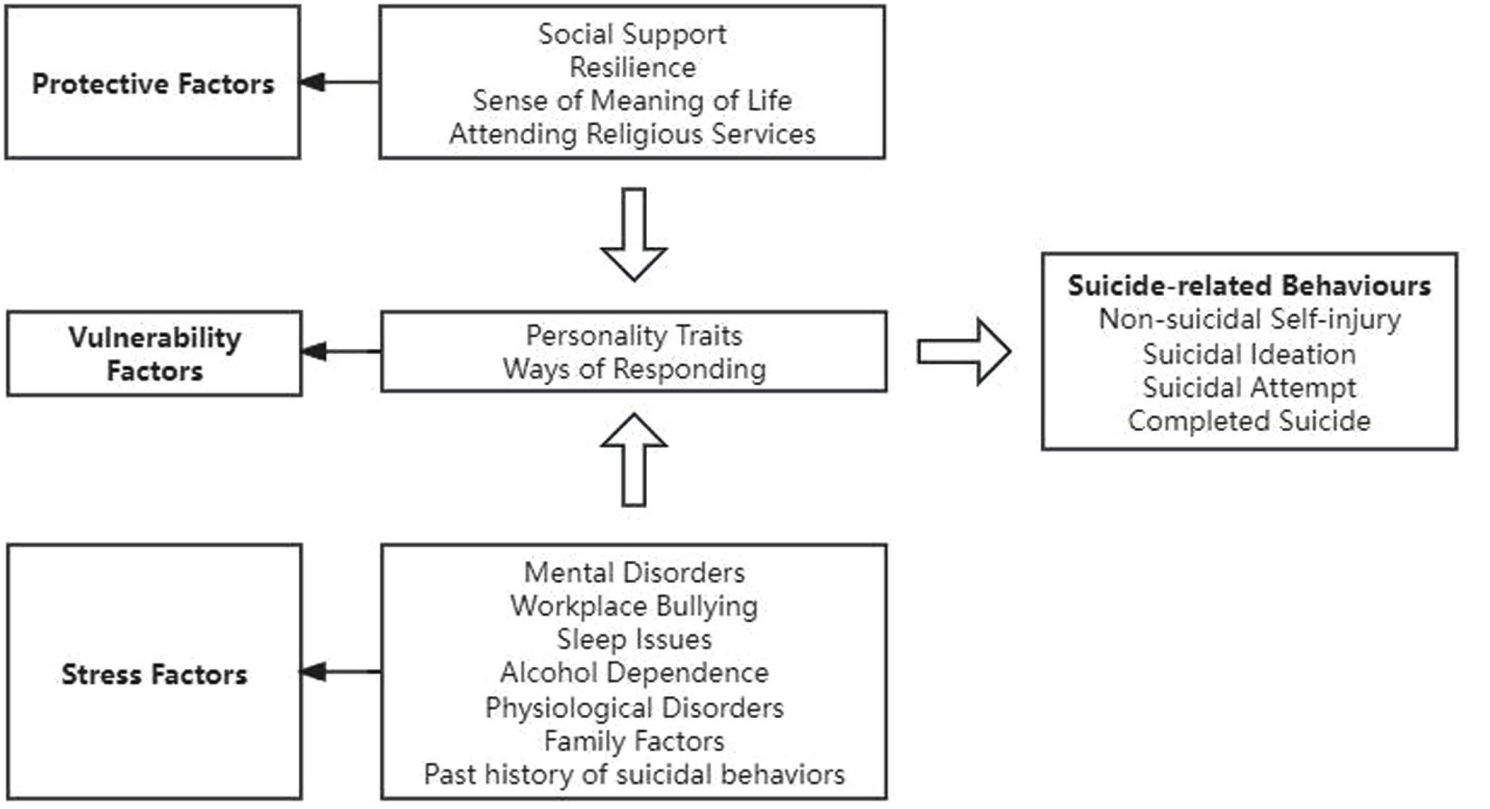
Factors of suicide-related behaviors based on stress-vulnerability model.

The “stress-vulnerability” model posits that individuals may develop illnesses as a result of the interplay between inherent predispositions and environmental influences, which subsequently manifest in varied behaviors (Mann et al., 1999). This theoretical framework suggests that suicidal behavior arises from the interaction of stressors and individual characteristics. In this context, “stress” is defined as a heightened state of tension triggered by perilous or unforeseen changes in one’s external environment. Individuals who encounter stressful events may be at an increased risk for psychological disorders, including mental illness and adverse emotional states. Conversely, refers to enduring susceptibilities or intrinsic traits that predispose individuals to suicidal behavior, such as impulsivity and psychoticism. Historically, the prevailing belief was that suicide resulted from specific stressors or mental health conditions. However, this model emphasizes the significance of the interaction among predispositions, psychological factors, and environmental influences, thereby offering a more holistic and innovative perspective for the identification, timely intervention, and prevention of suicide. Consequently, this study aims to investigate the factors influencing suicide-related behaviors based on this model. Figure 2 displays the factors that influence nurses’ suicide-related behaviors based on the stress-vulnerability model.

### 4.2 Vulnerability factors

#### 4.2.1 Personality traits

Personality is a persistent and stable psychological characteristic that gradually evolves, mediated by education and environment, based on biological heritage. Personality traits can influence an individual’s vulnerability and susceptibility to different sources of stress. A systematic review suggests that neuroticism, and extroverted personality traits are most strongly associated with suicide risk (Brezo et al., 2006). A 14-year study that followed individuals over time discovered a negative correlation between extroverted personality traits and suicide deaths (Maser et al., 2002). Other personality traits that are associated with suicide risk include aggression, impulsivity, and paranoia (Brezo et al., 2006). Personality traits are a fundamental factor in suicide, and the study of the relationship between personality traits and suicide is a deep and widely discussed topic.

#### 4.2.2 Coping strategy

The study has demonstrated a negative correlation between suicide and positive coping strategies, as well as a positive correlation between suicide and negative coping strategies (Hafferty et al., 2019). The findings of a cross-sectional study revealed that compared to nurses without suicidal ideation, nurses with suicidal ideations exhibit reluctance to seek assistance (Kelsey et al., 2021). Positive coping strategies, such as engaging in problem-solving and seeking social support, can alleviate the stress experienced by individuals and help alleviate unpleasant feelings. Negative coping strategies, such as fantasizing and withdrawal, do not promote effective problem-solving. When problems remain unresolved, individuals may experience a sense of hopelessness regarding their current circumstances and face an increased risk of suicidal behavior (Hafferty et al., 2019).

### 4.3 Stressor factors

#### 4.3.1 Mental disorders

Suicide is strongly associated with mental disorders, as 90% of suicide deaths occur in individuals who were diagnosed with a mental disorder at the time of their death. Common mental disorders linked to suicide include depression, schizophrenia, mania, anxiety disorders, and substance use disorders. Research has indicated that nurses who test positive for mental disorders have a notably increased occurrence of suicide-related behaviors. Nurses are more prone to experiencing depression and anxiety disorders (Freire et al., 2020; Groves et al., 2024). Individuals with depression often experience persistent negative emotions, impaired social functioning, diminished sense of belonging and burden in interpersonal relationships, as well as an increased risk of suicidal ideation. Moreover, they tend to employ maladaptive coping strategies when faced with stressful events, further elevating the likelihood of suicidal behavior. Meanwhile, individuals with anxiety may exhibit heightened sensitivity to stress and a more fragile emotional state. In the presence of intense negative emotions, they are at greater risk of impulsive actions leading to suicide (Freire et al., 2020).

Stress, burnout, and medical errors can indirectly contribute to nurses’ suicide-related behaviors through the manifestation of anxiety and depression (Kabir et al., 2023; Liu et al., 2023; Wang et al., 2023; Zhang et al., 2024). Excessive stress is linked to changes and irregularities in specific brain areas’ structure, function, and connectivity. Additionally, there is a consistent and meaningful positive relationship between these brain abnormalities and the intensity of depressive symptoms (Cheung and Yip, 2016). Nurses experiencing burnout often encounter emotional exhaustion, exhibit emotional detachment from their patients, and fail to acknowledge the significance and fulfillment of nursing work, potentially leading to depressive symptoms (Liu et al., 2023). Nurses often have persistent psychological trauma following adverse events, which can manifest as many psychological issues, including depression, anxiety, and post-traumatic stress disorder (Wang et al., 2023).

#### 4.3.2 Workplace bullying

Workplace bullying constitutes a pattern of psychological and relational aggression, systematically and persistently perpetrated by one or more individuals, resulting in the exclusion of the victim from the workplace. Research has indicated that there is a direct correlation between the number of different forms of bullying that a nurse encounters and the increased likelihood of suicide (Kabir et al., 2023; Lu et al., 2023). Workplace bullying can lead to nurses feeling alone and socially excluded, which has been identified as a strong predictor of suicide risk. Nurses who are subjected to workplace bullying may resort to unhealthy coping strategies such as substance abuse or alcohol misuse, thereby increasing the risk of suicide. Furthermore, nurses who experience bullying in a tense work environment are susceptible to work-related stress, which further elevates the risk of suicide (Lu et al., 2023).

#### 4.3.3 Sleep issues

Previous research has demonstrated that sleep issues, such as insomnia and poor sleep quality, represent distinct risk factors for suicidal behavior. The greater the number of sleep problems experienced by nurses, the increased likelihood of suicide risk they face (Mao et al., 2024; Wang et al., 2020). Sleep disorders can disrupt an individual’s emotional regulation, potentially triggering suicidal ideations. Additionally, cognitive impairments resulting from sleep disorders may contribute to increased risky and impulsive behaviors, ultimately leading to suicide. Furthermore, sleep disturbances have been identified as potential causes of mental illness, leading to decreased ability to handle stress, weakened immunological and cognitive performance, and heightened emotional distress (Wang et al., 2022).

#### 4.3.4 Alcohol dependence

Nurses often resort to maladaptive coping mechanisms, such as alcohol dependency, as a result of the stressful job conditions they face. Research has substantiated that nurses who are dependent on alcohol are more likely to have suicidal tendencies (Cheung and Yip, 2016; Groves et al., 2024). Alcohol use significantly increases the likelihood of suicide, with a mortality and suicide rate of 18 percent throughout a person’s lifetime (Sher and Zalsman, 2005). Alcohol dependence can result in a lack of behavioral inhibition among nurses, leading to impulsive behaviors and an increased risk for the nursing profession. Furthermore, chronic alcohol dependence can lead to specific psychological disorders, including but not limited to depression, hallucinations, paranoia, and may drive individuals to contemplate suicide as a coping mechanism.

#### 4.3.5 Physical disorders

Research has established a correlation between physical disorders and suicide-related behaviors among nurses. Physical disorders associated with nursing work encompass musculoskeletal discomfort, infectious ailments, reproductive system disorders, varicose veins, digestive system disorders, and cancer (Barnes et al., 2023; Cheung et al., 2016; Cheung and Yip, 2016; James et al., 2023; Xu et al., 2021). Nurses who have physical diseases often experience heightened apprehension and anxiety regarding the etiology, diagnosis, treatment, and prognosis of their condition. The decline in physical health due to physical disorders, accompanied by impaired functioning and loss of ability, significantly impacts the physical and mental well-being of nurses. Moreover, there is a significant positive genetic correlation between physical illnesses and symptoms of depression and anxiety, which in turn increases the risk of suicide.

#### 4.3.6 Family factors

Studies have substantiated that nurses who reside alone, are divorced, lack a partner, or have strained familial ties are more susceptible to suicide (Alyahya et al., 2023; Cheung and Yip, 2016; Freire et al., 2020; Stelnicki et al., 2020; Xu et al., 2021). Nurses who experience significant work–family conflicts may have family crises, resulting in a diminished quality of family life and happiness. These conflicts can also impact the quality and stability of marriages, leading to sadness, depression, and potential suicidal ideations. In a cross-sectional study conducted by Ding et al., the researchers utilized suicidal ideation screening questions and the Work–Family Conflict Scale to determine that work–family conflict is a significant risk factor for both lifetime and 1-year suicides (Ding et al., 2023). The significance of family crises in the emergence of suicide-related behaviors cannot be disregarded, and the tension between job and family responsibilities can frequently act as a catalyst for suicide.

Having a family history of suicide has always been acknowledged as a significant risk factor (Cheung and Yip, 2016). Research has shown that the children of parents who die by suicide are at a greater risk compared to persons without such exposure. A prospective study revealed that offspring were roughly five times more likely to attempt suicide if their parents had previously attempted suicide. The transmission of traits from parent to child may be associated with genetic or environmental factors. A comprehensive analysis indicates that being exposed to environments where suicide is prevalent significantly raises the likelihood of both suicide and suicide attempts.

#### 4.3.7 Past history of suicidal behaviors

A history of suicidal conduct is a significant factor that increases the chance of suicide (Freire et al., 2020; Höller and Forkmann, 2022). Prior research has indicated that a minimum of 50 percent of those who die by suicide have previously made at least one suicide attempt. Multiple instances of suicide attempts were correlated with elevated rates of mental disease and heightened degrees of sadness. Thus, a previous record of suicidal activity serves as a risk factor for suicide-related behavior among nurses.

### 4.4 Protective factors

#### 4.4.1 Social support

Social support encompasses the diverse array of assistance and aid individuals obtain from their social connections. Prior research indicates that social support is vital in mitigating the likelihood of suicide-related behaviors among nurses (Groves et al., 2024; Kantorski et al., 2022; Xu et al., 2021). Liu et al. utilized the Suicide Ideation Screening Questionnaire and the Perceived Social Support Scale (PSSS) to conduct a survey on 3,426 medical workers in China regarding their suicide ideation and social support. The study revealed that social support exerts a direct influence on suicide ideation (Liu et al., 2021). Receiving increased levels of social support can contribute to the reduction of nurses’ substance abuse, work–family conflict, workplace bullying, and depressive symptoms, thereby lowering the incidence of nurse suicide (Wang et al., 2018). Proposes that nurses can mitigate their risk of suicide by receiving increased levels of social support.

#### 4.4.2 Resilience

Psychological resilience refers to an individual’s capacity to maintain or regain psychological well-being in adversity or stress. Suicidal ideation is linked to dysregulation of the neuroendocrine pathway, whereas psychological resilience is associated with the stability of the neuroendocrine pathway. Meanwhile, psychological resilience can prevent individuals from resorting to suicide as a means of escaping difficulties by bolstering their capacity to adapt and cope with challenges, fostering positive self-appraisals, and enhancing coping skills (Shi and Jiang, 2014). Furthermore, Schierberl’s research findings suggest a positive association between the psychological resilience of nurses and their social support, which also acts as a moderator for depression and anxiety (Schierberl et al., 2021).

#### 4.4.3 Sense of meaning of life

Meaning in life refers to an individual’s capacity to choose the direction and objective of their current or future life by reflecting on the reason or purpose of their existence. Individuals experiencing a lack of existential meaning are more likely to succumb to stress and exhibit negative emotions such as depression, anxiety, and even suicidal attempt. A high level of life meaning can enhance positive emotions such as well-being and satisfaction in individuals, and also plays a crucial role in coping with stress and promoting post-traumatic growth. The perception of life’s meaning has been demonstrated to be a potent factor in mitigating depression and suicidal ideation (Sun et al., 2022).

#### 4.4.4 Attending religious services

It is essential to exercise caution when evaluating the protective role of religion or spiritual beliefs about suicide. Belief itself may exert a protective influence due to its provision of a structured belief system and encouraging healthy behaviors (Cheung et al., 2016). However, numerous religious and cultural beliefs and practices involve moral judgments about suicide, which could exacerbate an individual’s feelings of shame and stigma related to suicide, thereby hindering their help-seeking behavior. The protective value of religion and spiritual beliefs may lie in fostering a community with shared values, social cohesion, and mutual support. Nkansah-Amankra et al. discovered that regular church attendance and participation in weekend church prayers reduced suicidal ideation by 42% (Nkansah-Amankra et al., 2012). Therefore, while religion and spiritual beliefs may offer some protection against suicide, this depends on the specific culture, practices, and interpretations within a particular context.

## 5 Strategies for tertiary suicide prevention among nurses

### 5.1 Overview of tertiary suicide prevention

The main focus of suicide intervention is prevention, and in the field of public health, a tertiary prevention model is commonly employed to develop strategies for preventing and treating diseases. Tertiary prevention involves using health promotion strategies to mitigate risk factors and enhance protective factors against illness, including suicide prevention. This extends to the tertiary prevention of suicide. Presently, research has implemented the three tiers of suicide prevention in the general population, teenagers, and patients, respectively. In all cases, the efficacy of these approaches has been proven. Table 2 displays the strategies applied for the tertiary prevention of suicide among nurses.

**Table 2:** Strategies for tertiary prevention of suicide among nurses.

Author(s)	Country	Methods	Population	Strategies
Primary prevention strategies				
Lascelles (2024)	England	Review	Nurse	Educate nurses about mental health and well-being A culture of safety and well-being Information about support for mental health difficulties and problem at substance use
Groves et al. (2023)	England	Systematic review	Nurse	Education about physical health problems, workplace wellbeing, substance misuse and mental health problems. Peer-support to foster social support and integration
Lange (2023)	USA	Review	Nurse	Education on suicide prevention for nurses Decreasing shift work Providing education and resources to decrease or prevent workplace bullying
Kabir et al. (2023)	Bangladesh	Cross-sectional study	Nurse	Make a favorable work environment
Barnes et al., 2023	England	Qualitative research	Nurse	Educate nurses reducing the stigmatization of mental health problems and removing barriers to help-seeking behaviors
Lee and Friese, 2021	USA	Review	Nurse	Education to lower the stigma of seeking help Challenges in equitable workloads, adequate physical and human resources
Wang et al. (2020)	China	Cross-sectional study	Nurse	Decreasing shift work
Stagg et al. (2011)	USA	Experimental study	Nurse	Cognitive rehearsal program
Secondary prevention strategies				
Lascelles (2024)	England	Review	Nurse	Healer Education Assessment and Referral (HEAR) program
Groves et al. (2023)	England	Systematic review	Nurse	Suicide risk screening
Lange (2023)	USA	Review	Nurse	Healer Education Assessment and Referral (HEAR) program MINDBODYSTRONG (a cognitive behavioral therapy-based approach)
Hofstetter and Mayer (2023)	USA	Review	Nurse	Healer Education Assessment and Referral (HEAR) program
Alyahya et al. (2023)	Saud	Cross-sectional study	Nurse	A free, easily accessible confidential suicide prevention hotline
Davis et al. (2021)	USA	Retrospective cohort study	Nurse	Healer Education Assessment and Referral (HEAR) program
Lee and Friese (2021)	USA	Review	Nurse	Screening to identify nurses at suicide risk
Dutheil et al. (2019)	Japan	Systematic review and meta-analysis	Nurse	Screening, assessment, and referral
Cheung and Yip (2016)	China	Cross-sectional study	Nurse	A continuous monitoring system in the healthcare setting detecting and managing the risks of self-harm in nurses
Tertiary prevention strategies				
Lascelles et al. (2024)	England	Review	Nurse	Follow-up support (phone calls, crisis cards, mails, postal cards.)
Hofstetter and Mayer (2023)	USA	Review	Nurse	Providing Suicide and Crisis Lifeline and Suicide Prevention Resource Center

### 5.2 Primary prevention strategies

In the primary prevention strategy for suicide among nurses, various measures can be implemented to target both protective and risk factors. These measures include enhancing peer support to improve nurses’ social support and sense of integration, implementing rational shift scheduling to safeguard nurses’ rest and sleep time, and addressing workplace bullying, substance abuse, and mental disorders through education programs (Barnes et al., 2023; Groves et al., 2023; Lange, 2023; Lascelles et al., 2024; Lee and Friese, 2021; World Health Organization, 2018). Additionally, reducing access to means of suicide, raising suicide awareness and publicity, and implementing preventive measures are crucial components of this strategy (Kabir et al., 2023; Lee and Friese, 2021; Wang et al., 2020). Furthermore, pertinent research has demonstrated that engaging in cognitive exercises might enhance nurses’ awareness of workplace bullying and equip them with adequate coping strategies to reduce the likelihood of suicide.

### 5.3 Secondary prevention strategies

Recent research indicates that the utilization of screening and assessment tools for suicide risk, in conjunction with intervention measures, is both practical and has the potential to save lives (Alyahya et al., 2023; Cheung and Yip, 2016; Dutheil et al., 2019; Groves et al., 2023; Lee and Friese, 2021; World Health Organization, 2018). Aside from commonly used screening tools like the Adult Suicidal Ideation Questionnaire, Columbia Suicide Severity Rating Scale (C-SSRS), and other scales that assess suicidal ideation and behavior, suicide-related items in other scales, such as those that measure depression and despair, can also be utilized for screening purposes. The assessment of suicide risk in nurses is conducted through a combination of screening, observation, assessment, and interviews. Two commonly used suicide risk assessment tools are the Suicide Assessment Five-step Evaluation and Triage (SAFE-T) and prevention-oriented risk formulation.

Interventions should be promptly implemented for nurses at risk of suicide. Crisis intervention methods, such as cognitive behavioral therapy, mindfulness-based stress reduction, and MINDBODYSTRONG (MBS), can be utilized to manage negative emotions and mitigate their suicide risk. Studies have demonstrated that the MBS program effectively alleviates nurses’ anxiety, depression, and burnout while enhancing job satisfaction and overall well-being. A randomized controlled trial has shown that the HEAR program effectively identifies at-risk nurses and facilitates their referral to mental health care (Davis et al., 2021; Lange, 2023; Lascelles et al., 2024).

### 5.4 Tertiary prevention strategies

Individuals who have attempted suicide are at a high risk of experiencing repeated suicide-related behaviors. Sustained intervention and systematic follow-up by healthcare professionals are essential for mitigating repeated suicide attempts in this nurse population. Diverse approaches can be employed for conducting follow-ups with nursing staff while ensuring periodic reevaluation of suicide risk throughout the process. Empirical evidence supports the positive effectiveness of post-suicide patient monitoring. Additional tertiary prevention strategies encompass disseminating information about pertinent service organizations, bolstering social support networks, and regulating media portrayal of suicides (Hofstetter and Mayer, 2023; Lascelles et al., 2024; World Health Organization, 2018).

## 6 Discussion

Comprehensive prior investigations on suicide-related behaviors among nurses have demonstrated that the overall incidence of suicide-related behaviors among nurses is at a relatively high level. This study investigates nurse suicide using the “stress-vulnerability” model. It identifies the characteristics that make nurses more vulnerable to suicide, the stress factors that contribute to nurse suicide, and the protective factors that can help prevent nurse suicide-related behaviors. This study developed a tertiary prevention model for nursing suicide based on the tertiary prevention model for suicide.

The findings of this research reveal that the results of several studies on the incidence of suicidal behaviors among nurses differed greatly. The lifetime prevalence of suicidal ideation among nurses varies from 4.3 to 44.58%, with the majority falling within the range of 10–25%. Similarly, the lifetime prevalence of suicide attempts ranges from 2.9 to 12.6%, with the majority falling within the range of 8–10%. Compared to other groups, the prevalence of lifetime suicidal ideation and suicide attempts among adults is approximately 9.2 and 2.7%, respectively. Among university students globally, the lifetime incidence of suicidal ideations is 22.3%, whereas the frequency of suicide attempts is 3.2%. The above comparison demonstrates that nurses have a high risk of suicide.

This study provides a comprehensive review of the factors influencing nurse suicide based on the stress-vulnerability model. Personality traits and coping style are the vulnerability factors of suicide in nurses. It is worth noting that personality traits are stable and lasting, and personality traits have a certain impact on suicidal behaviors, but the effect is not large (Brezo et al., 2006). As a bad cognitive style, negative coping style can affect nurses’ cognitive ability of suicide, resulting in distorted understanding and suicidal behaviors (Hafferty et al., 2019). Therefore, to correct the bad coping style of nurses, we should actively guide the correct cognitive style of nurses. Different from the general population, the risk factors of suicide in nurses are also tied to their particular vocational features, such as job fatigue, medical blunders, work–family conflict and workplace violence. Nursing managers are advised to alleviate nurses’ workloads, assist in coordinating responses to familial conflicts, offer prompt psychological support to nurses facing stressful incidents such as medical errors, routinely evaluate nurses’ psychological issues and screen for suicidal ideation, and promptly engage mental health professionals for intervention in cases of suicide risk, thereby minimizing the potential for nurse suicides (Liu et al., 2021). In contrast to stress factors, exploring protective factors can provide intervention measures for nurse suicide; this study found that social support plays an essential role in protecting against nurse suicide risk. Current research findings indicate that the level of social support for nurses is moderate, with family support scoring higher than other dimensions of support. At present, most of the research on the protective factors of nurse suicide is also aimed at internal factors of the individual, few studies have explored the protective effects of sociocultural or collective level factors on suicide-related behaviors among nurses, and future research is needed.

The study provides an overview of prevention strategies for addressing nurses’ suicide. Specific primary prevention measures for the nursing population include cognitive training and optimizing shift schedules. Stagg et al.’s study enhanced nurses’ understanding, consciousness, and self-assurance regarding workplace bullying using a cognitive drill intervention program focused on 10 different workplace bullying scenarios (Stagg et al., 2011). The reorganization of shifts is more favorable for promoting relaxation and sleep among nurses while also decreasing their workload, work-related stress, and burnout (Kabir et al., 2023; Lee and Friese, 2021; Wang et al., 2020). Distinctive strategies for secondary prevention encompass the MBS and the HEAR program. MBS is a standardized program rooted in cognitive behavioral therapy, encompassing practical intervention measures for short-term and long-term health strategies and skills (Lange, 2023). Additionally, the HEALER Education Assessment & Referral (HEAR) program serves as a crucial secondary prevention measure by focusing on delivering psychological education, assessment, and referral services; the American Medical Association has endorsed it as a best practice for preventing suicide (Davis et al., 2021; Lange, 2023; Lascelles et al., 2024). At present, there are limited specific interventions for nurses to prevent suicide at the tertiary level; however, general measures such as analyzing and addressing the underlying causes of suicide and regularly reassessing the risk of suicide remain relevant to nurses.

Most suicide prevention measures for nurses focus on the nurses or hospital management, with few involving government or national organizations (García-Iglesias et al., 2022; Lascelles et al., 2024). Given the high suicide risk among nurses, it is essential to establish a suicide prevention mechanism that includes government guidance and interdepartmental cooperation. A dedicated organization involving nurses, their families, medical facilities, and social organizations can effectively reduce suicidal behaviors. The government should also enhance nurses’ rights through relevant laws and regulations, while hospital management should implement measures to address potential risks during diagnosis and hospitalization (Basu et al., 2023).

There are limited studies on nurse suicide. The authors suggest several areas for further research: First, there is a lack of demographic data regarding the occurrence of suicide-related behaviors among nurses, including factors such as gender, age, and professional title. Second, cultural aspects are rarely acknowledged in both domestic and international research. Third, the current research on the factors contributing to suicide vulnerability among nurses has not adequately addressed the profound psychological and social influences, including cognitive impairment, memory bias, decision-making difficulties, subjective distress, and social distortion. Consequently, future studies should focus on a more comprehensive and in-depth exploration of these factors. Fourth, the interaction between vulnerability factors and stressors needs to be explored. Fifth, there is a limited understanding of the diversity among nurses, including geographical differences and the experiences of nurses at various levels within hospitals. Additionally, there are no large-scale studies examining suicide-related issues among community nurses. Increasing awareness, accelerating research development, and implementing effective intervention strategies to prevent suicide-related behaviors among nurses are shared objectives for both theoretical studies and practical applications in the field of nurse suicide prevention in the future.

This paper presents a comprehensive and standardized review of the current state, influencing factors, and prevention strategies related to suicidal behaviors among nurses, based on the stress-vulnerability model and the tertiary prevention model. By thoroughly examining the suicidal behaviors of nurses, this study aims to enhance societal and medical industry awareness of the mental health challenges faced by nurses, thereby reducing misunderstandings and prejudices surrounding their suicidal behaviors. The in-depth discussion of the stress-vulnerability model enriches its application within the nursing field and offers new ideas and methods for preventing suicidal behaviors among nurses. Additionally, the review of prevention strategies emphasizes the necessity of implementing tailored measures at various levels, providing a scientific foundation for relevant departments and medical institutions to develop targeted intervention strategies.

### 6.1 Limitations

The study is subject to certain limitations: (1) The meaning of the term “nurse” is ambiguous. Implementing a universally accepted definition of “nurse” would enhance the capacity to compare research findings. 2) The search scope is limited to academic articles published in the literature, excluding any relevant data from external sources. (3) The review also omitted papers that were not written in English or Chinese, perhaps resulting in the deletion of other important works.

## 7 Conclusion

This paper offers an extensive review of the current status, factors that influence, and prevention strategies of nurses’ suicide-related behaviors based on the stress-vulnerability model and the tertiary prevention model of suicide. It aims to provide guidance for the management of nurses’ suicide. Nurses are an essential part of the healthcare system, and the prevention of nurse suicide-related behaviors is of great significance in maintaining the stable operation of healthcare institutions, safeguarding the medical safety of patients, and respecting the professional values of nurses.

## References

[ref1] Alyahya K. I. Alrefaei R. M. Almadhyani L. F. AlQuwayz S. S. AlOmairini M. I. Alsayed F. A.. (2023). The prevalence and correlation of suicidal ideation among nurses in King Saud University Medical City. Cureus 15:e44859. doi: 10.7759/cureus.44859, PMID: 37809273 PMC10560092

[ref2] Badrfam R. Zandifar A. Khonsari N. M. Qorbani M. (2023). Suicidal ideation, burnout, and their correlation among health care workers at the end of the fourth wave of the COVID-19 pandemic in Alborz Province, Iran. Front. Psychiatry 14:1261105. doi: 10.3389/fpsyt.2023.1261105, PMID: 37900293 PMC10603268

[ref3] Barnes A. Ye G. Y. Ayers C. Choflet A. Lee K. C. Zisook S.. (2023). Entangled: a mixed method analysis of nurses with mental health problems who die by suicide. Nurs. Inq. 30:e12537. doi: 10.1111/nin.12537, PMID: 36283975

[ref4] Basu N. Barinas J. Williams K. Clanton C. Smith P. N. (2023). Understanding nurse suicide using an ideation-to-action framework: an integrative review. J. Adv. Nurs. 79, 4472–4488. doi: 10.1111/jan.15681, PMID: 37278387

[ref5] Bonner R. L. Rich A. R. (1988). A prospective investigation of suicidal ideation in college students: a test of a model. Suicide Life Threat. Behav. 18, 245–258. doi: 10.1111/j.1943-278x.1988.tb00160.x 3188140

[ref6] Brady C. Fenton C. Loughran O. Hayes B. Hennessy M. Higgins A.. (2022). Nursing home staff mental health during the Covid-19 pandemic in the Republic of Ireland. Int. J. Geriatr. Psychiatry 37. doi: 10.1002/gps.5648, PMID: 34729818 PMC8646737

[ref7] Brady C. Shackleton E. Fenton C. Loughran O. Hayes B. Hennessy M.. (2023). Worsening of mental health outcomes in nursing home staff during the COVID-19 pandemic in Ireland. PLoS One 18:e0291988. doi: 10.1371/journal.pone.0291988, PMID: 37751434 PMC10521981

[ref8] Brezo J. Paris J. Turecki G. (2006). Personality traits as correlates of suicidal ideation, suicide attempts, and suicide completions: a systematic review. Acta Psychiatr. Scand. 113, 180–206. doi: 10.1111/j.1600-0447.2005.00702.x 16466403

[ref9] Cheung T. Lee P. H. Yip P. S. (2016). Suicidality among Hong Kong nurses: prevalence and correlates. J. Adv. Nurs. 72, 836–848. doi: 10.1111/jan.12869, PMID: 26711369

[ref10] Cheung T. Yip P. S. (2016). Self-harm in nurses: prevalence and correlates. J. Adv. Nurs. 72, 2124–2137. doi: 10.1111/jan.12987 27121340

[ref11] Chin W. S. Chen Y. C. Ho J. J. Cheng N. Y. Wu H. C. Shiao J. (2019). Psychological work environment and suicidal ideation among nurses in Taiwan. J. Nurs. Scholarsh. 51, 106–113. doi: 10.1111/jnu.12441, PMID: 30466180

[ref12] Dalum H. S. Hem E. Ekeberg O. Reneflot A. Stene-Larsen K. Hauge L. J. (2024). Suicide rates among health-care professionals in Norway 1980-2021. J. Affect. Disord. 355, 399–405. doi: 10.1016/j.jad.2024.03.128, PMID: 38537752

[ref13] Davidson J. E. Proudfoot J. Lee K. Zisook S. (2019). Nurse suicide in the United States: analysis of the Center for Disease Control 2014 National Violent Death Reporting System dataset. Arch. Psychiatr. Nurs. 33, 16–21. doi: 10.1016/j.apnu.2019.04.006, PMID: 31711588 PMC7927355

[ref14] Davidson J. E. Stuck A. R. Zisook S. Proudfoot J. (2018). Testing a strategy to identify incidence of nurse suicide in the United States. J. Nurs. Adm. 48, 259–265. doi: 10.1097/NNA.0000000000000610, PMID: 29672372

[ref15] Davis M. A. Cher B. A. Y. Friese C. R. Bynum J. P. W. (2021). Association of US nurse and physician occupation with risk of suicide. JAMA Psychiatry 78, 651–658. doi: 10.1001/jamapsychiatry.2021.0154, PMID: 33851982 PMC8047773

[ref16] Ding Y. Sun L. Gui Z. Wang K. Li X. (2023). Association of work-family conflict with suicidal ideation among medical staff:a cross-sectional survey in Shandong province. China J. Public Health 39, 1250–1254. doi: 10.11847/zgggws1141449

[ref17] Dutheil F. Aubert C. Pereira B. Dambrun M. Moustafa F. Mermillod M.. (2019). Suicide among physicians and health-care workers: a systematic review and meta-analysis. PLoS One 14:e226361. doi: 10.1371/journal.pone.0226361, PMID: 31830138 PMC6907772

[ref18] Fen T. Deying H. Yi Z. (2019). Suicide terminology classification universal precautions review. Chin. J. Nurs. 54, 1740–1745. doi: 10.3761/j.issn.0254-1769.2019.11.029

[ref19] Feskanich D. Hastrup J. L. Marshall J. R. Colditz G. A. Stampfer M. J. Willett W. C.. (2002). Stress and suicide in the Nurses' health study. J. Epidemiol. Community Health 56, 95–98. doi: 10.1136/jech.56.2.95, PMID: 11812806 PMC1732078

[ref20] Freire F. O. Marcon S. R. Espinosa M. M. Santos H. Kogien M. Lima N.. (2020). Factors associated with suicide risk among nurses and physicians: a cross-section study. Rev. Bras. Enferm. 73Suppl 1:e20200352. doi: 10.1590/0034-7167-2020-0352 33084840

[ref21] García-Iglesias J. J. Gómez-Salgado J. Fernández-Carrasco F. J. Rodríguez-Díaz L. Vázquez-Lara J. M. Prieto-Callejero B.. (2022). Suicidal ideation and suicide attempts in healthcare professionals during the COVID-19 pandemic: a systematic review. Front. Public Health 10:1043216. doi: 10.3389/fpubh.2022.1043216, PMID: 36561871 PMC9767440

[ref22] Gonzalez Mendez M. J. Ma L. Alvarado R. Ramirez J. Xu K. Xu H.. (2022). A multi-center study on the negative psychological impact and associated factors in Chinese healthcare workers 1 year after the COVID-19 initial outbreak. Int. J. Public Health 67. doi: 10.3389/ijph.2022.1604979, PMID: 36090824 PMC9454095

[ref23] Groves S. Lascelles K. Bale L. Brand F. Casey D. Hawton K. (2024). Self-harm by nurses and midwives - a study of hospital presentations. Crisis 45, 128–135. doi: 10.1027/0227-5910/a000936, PMID: 38234244 PMC10985583

[ref24] Groves S. Lascelles K. Hawton K. (2023). Suicide, self-harm, and suicide ideation in nurses and midwives: a systematic review of prevalence, contributory factors, and interventions. J. Affect. Disord. 331, 393–404. doi: 10.1016/j.jad.2023.03.027 36933670

[ref25] Hafferty J. D. Navrady L. B. Adams M. J. Howard D. M. Campbell A. I. Whalley H. C.. (2019). The role of neuroticism in self-harm and suicidal ideation: results from two UK population-based cohorts. Soc. Psychiatry Psychiatr. Epidemiol. 54, 1505–1518. doi: 10.1007/s00127-019-01725-7, PMID: 31123787 PMC6858388

[ref26] Hamza C. A. Stewart S. L. Willoughby T. (2012). Examining the link between nonsuicidal self-injury and suicidal behavior: a review of the literature and an integrated model. Clin. Psychol. Rev. 32, 482–495. doi: 10.1016/j.cpr.2012.05.003 22717336

[ref27] Hofstetter T. Mayer N. L. (2023). CE: suicide prevention: protecting the future of nurses. Am. J. Nurs. 123, 30–36. doi: 10.1097/01.NAJ.0000996556.74490.80 37934871

[ref28] Höller I. Forkmann T. (2022). Ambivalent heroism? - Psychological burden and suicidal ideation among nurses during the Covid-19 pandemic. Nurs. Open 9, 785–800. doi: 10.1002/nop2.1130, PMID: 34792286 PMC8661563

[ref29] Hong S. Ai M. Xu X. Wang W. Chen J. Zhang Q.. (2021). Immediate psychological impact on nurses working at 42 government-designated hospitals during COVID-19 outbreak in China: a cross-sectional study. Nurs. Outlook 69, 6–12. doi: 10.1016/j.outlook.2020.07.007, PMID: 32919788 PMC7368912

[ref30] James K. E. Agarwal S. Armenion K. L. Clapp C. Barnes A. Ye G. Y.. (2023). A deductive thematic analysis of nurses with job-related problems who completed suicide during the early COVID-19 pandemic: a preliminary report. Worldviews Evid.-Based Nurs. 20, 96–106. doi: 10.1111/wvn.12640, PMID: 36991524

[ref31] Kabir H. Chowdhury S. R. Roy A. K. Chowdhury S. A. Islam M. N. Chomon R. J.. (2023). Association of workplace bullying and burnout with nurses' suicidal ideation in Bangladesh. Sci. Rep. 13:14641. doi: 10.1038/s41598-023-41594-4, PMID: 37669987 PMC10480219

[ref32] Kantorski L. P. de Oliveira M. M. Dos Santos Treichel C. A. Bakolis I. Alves P. F. Christello Coimbra V. C.. (2022). Mental health of nursing professionals during the COVID-19 pandemic: a cross-sectional study. Rev. Saude Publica 56, 1–8. doi: 10.11606/s1518-8787.2022056004122, PMID: 35293941 PMC8910133

[ref33] Kelsey E. A. West C. P. Cipriano P. F. Peterson C. Satele D. Shanafelt T.. (2021). Original research: suicidal ideation and attitudes toward help seeking in U.S. nurses relative to the general working population. Am. J. Nurs. 121, 24–36. doi: 10.1097/01.NAJ.0000798056.73563.fa, PMID: 34629376

[ref34] Lange M. (2023). The hidden crisis of nurse suicide. Nursing 53, 28–32. doi: 10.1097/01.NURSE.0000978856.91159.41, PMID: 37856296

[ref35] Lascelles K. Groves S. Hawton K. (2024). Suicide among nurses: what can we do to protect our workforce? J. Adv. Nurs. 80, 1667–1669. doi: 10.1111/jan.15956 37950401

[ref36] Lee K. A. Friese C. R. (2021). Deaths by suicide among nurses: a rapid response call. J. Psychosoc. Nurs. Ment. Health Serv. 59, 3–4. doi: 10.3928/02793695-20210625-01, PMID: 34343054 PMC8344804

[ref37] Liu H. Sun L. Liu T. Wang H. Gui Z. (2021). Prevalence and correlations of suicidal ideation among medical staff in general hospitals. Chin. Ment. Health J. 35, 389–394. doi: 10.3969/j.issn.1000-6729.2021.05.007

[ref38] Liu G. Tong Y. Li J. Sun X. Chen L. Zheng X.. (2023). Burnout, moral injury, and suicidal/self-harm ideation among healthcare professionals in mainland China: insights from an online survey during the COVID-19 pandemic. Int. J. Psychiatry Med. 59, 487–502. doi: 10.1177/00912174231219041, PMID: 38047438

[ref39] Lu Y. Sun M. Li Y. Wu L. Zhang X. Wang J.. (2023). Association of workplace bullying with suicide ideation and attempt among Chinese nurses during the COVID-19 pandemic. J. Clin. Psychol. Med. Settings 30, 687–696. doi: 10.1007/s10880-022-09915-3, PMID: 36272037 PMC9589744

[ref40] Mann J. J. Waternaux C. Haas G. L. Malone K. M. (1999). Toward a clinical model of suicidal behavior in psychiatric patients. Am. J. Psychiatry 156, 181–189. doi: 10.1176/ajp.156.2.181, PMID: 9989552

[ref41] Mao F. Wan J. Sun Y. Yang B. Wang Y. Cao F. (2024). Association between transition patterns of sleep problems and suicidal ideation in Chinese female nurses: a prospective study. J. Clin. Psychol. 80, 279–290. doi: 10.1002/jclp.23612, PMID: 37847787

[ref42] Martinez-Arriaga R. J. Dominguez-Rodriguez A. Erika Herdoiza-Arroyo P. Robles-Garcia R. de la Rosa-Gomez A. Gonzalez A. F.. (2023). Suicide risk and associated factors in healthcare workers seeking psychological support during COVID-19: a cross-sectional study. Psychol. Health Med. 28, 3076–3090. doi: 10.1080/13548506.2023.2216469, PMID: 37224286

[ref43] Maser J. D. Akiskal H. S. Schettler P. Scheftner W. Mueller T. Endicott J.. (2002). Can temperament identify affectively ill patients who engage in lethal or near-lethal suicidal behavior? A 14-year prospective study. Suicide Life Threat. Behav. 32, 10–32. doi: 10.1521/suli.32.1.10.22183, PMID: 11931008

[ref44] Merkle S. L. Welton M. van Zyl A. Chong M. Tanner A. Rose C. E.. (2023). Symptoms of depression, anxiety, and post-traumatic stress disorder, and suicidal ideation among school nurses in prekindergarten through grade 12 schools - United States, march 2022. J. Sch. Nurs. 39, 114–124. doi: 10.1177/10598405221131048, PMID: 36315836 PMC9988285

[ref45] Nkansah-Amankra S. Diedhiou A. Agbanu S. K. Agbanu H. L. Opoku-Adomako N. S. Twumasi-Ankrah P. (2012). A longitudinal evaluation of religiosity and psychosocial determinants of suicidal behaviors among a population-based sample in the United States. J. Affect. Disord. 139, 40–51. doi: 10.1016/j.jad.2011.12.027, PMID: 22483954

[ref46] Patrician P. A. Peterson C. McGuinness T. M. (2020). Original research: suicide among RNs: an analysis of 2015 data from the National Violent Death Reporting System. Am. J. Nurs. 120, 24–28. doi: 10.1097/01.NAJ.0000718624.25806.3f, PMID: 32976149 PMC7552572

[ref47] Peng P. Chen Q. Liang M. Liu Y. Chen S. Wang Y.. (2022). A network analysis of anxiety and depression symptoms among Chinese nurses in the late stage of the COVID-19 pandemic. Front. Public Health 10:996386. doi: 10.3389/fpubh.2022.996386, PMID: 36408014 PMC9667894

[ref4000] Pede S. Uguccioni M. (2001). AHCPR/AHRQ guidelines. Agency for Health Care Policy and Research and Agency for Health Care Research and Quality. Ital Heart J 2 Suppl. 1, 60–68., PMID: 11347031

[ref48] Peters M. D. J. Marnie C. Tricco A. C. Pollock D. Munn Z. Alexander L.. (2020). Updated methodological guidance for the conduct of scoping reviews. JBI Evid Synth 18, 2119–2126. doi: 10.11124/JBIES-20-00167, PMID: 33038124

[ref49] Rozanov V. Carli V. (2012). Suicide among war veterans. Int. J. Environ. Res. Public Health 9, 2504–2519. doi: 10.3390/ijerph9072504, PMID: 22851956 PMC3407917

[ref50] Salari N. Hosseinian-Far A. Jalali R. Vaisi-Raygani A. Rasoulpoor S. Mohammadi M.. (2020). Prevalence of stress, anxiety, depression among the general population during the COVID-19 pandemic: a systematic review and meta-analysis. Glob. Health 16:57. doi: 10.1186/s12992-020-00589-w, PMID: 32631403 PMC7338126

[ref51] Schierberl S. A. Ayotte B. J. Kellogg M. B. (2021). Moderating roles of resilience and social support on psychiatric and practice outcomes in nurses working during the COVID-19 pandemic. SAGE Open Nurs. 7:2085677397. doi: 10.1177/23779608211024213, PMID: 34189262 PMC8209788

[ref52] Scott R. Aboud A. Smith T. (2023). Using the stress-vulnerability model to better understand suicide in prison populations. Psychiatr. Psychol. Law 30, 299–333. doi: 10.1080/13218719.2021.2013340, PMID: 37346061 PMC10281367

[ref53] Sher L. Zalsman G. (2005). Alcohol and adolescent suicide. Int. J. Adolesc. Med. Health 17, 197–203. doi: 10.1515/ijamh.2005.17.3.197, PMID: 16231470

[ref54] Shi X. Jiang H. (2014). A review of psychological resilience and coping styles for nursing staff. J. Nurs. Sci. 29, 83–84. doi: 10.3870/hlxzz.2014.24.083

[ref55] Stagg S. J. Sheridan D. Jones R. A. Speroni K. G. (2011). Evaluation of a workplace bullying cognitive rehearsal program in a hospital setting. J. Contin. Educ. Nurs. 42, 395–403. doi: 10.3928/00220124-20110823-45, PMID: 21877661

[ref56] Stang A. (2010). Critical evaluation of the Newcastle-Ottawa scale for the assessment of the quality of nonrandomized studies in meta-analyses. Eur. J. Epidemiol. 25, 603–605. doi: 10.1007/s10654-010-9491-z, PMID: 20652370

[ref57] Stelnicki A. M. Jamshidi L. Angehrn A. Nicholas C. R. (2020). Suicidal behaviors among nurses in Canada. Can. J. Nurs. Res. 52, 226–236. doi: 10.1177/0844562120934237, PMID: 32552154

[ref58] Sucharew H. Macaluso M. (2019). Progress notes: methods for research evidence synthesis: the scoping review approach. J. Hosp. Med. 14, 416–418. doi: 10.12788/jhm.3248, PMID: 31251164

[ref59] Sun F. K. Wu M. K. Yao Y. Chiang C. Y. Lu C. Y. (2022). Meaning in life as a mediator of the associations among depression, hopelessness and suicidal ideation: a path analysis. J. Psychiatr. Ment. Health Nurs. 29, 57–66. doi: 10.1111/jpm.12739, PMID: 33559221

[ref60] Wang D. Chen H. Chen Z. Yang Z. Zhou X. Tu N.. (2022). Resilience buffers the association between sleep disturbance and psychotic-like experiences in adolescents. Schizophr. Res. 244, 118–125. doi: 10.1016/j.schres.2022.05.018, PMID: 35661549

[ref61] Wang J. Mann F. Lloyd-Evans B. Ma R. Johnson S. (2018). Associations between loneliness and perceived social support and outcomes of mental health problems: a systematic review. BMC Psychiatry 18:156. doi: 10.1186/s12888-018-1736-5, PMID: 29843662 PMC5975705

[ref62] Wang M. Wei Z. Wang Y. Sun L. (2023). Mediating role of psychological distress in the associations between medical errors, adverse events, suicidal ideation and plan among operating room nurses in China: a cross-sectional study. BMJ Open 13:e69576:e069576. doi: 10.1136/bmjopen-2022-069576, PMID: 37399442 PMC10314585

[ref63] Wang J. Zhang X. Yang B. Li J. Li Y. Chen Q.. (2020). Suicidal ideation among nurses: unique and cumulative effects of different subtypes of sleep problems. J. Affect. Disord. 276, 600–607. doi: 10.1016/j.jad.2020.07.095, PMID: 32871691

[ref64] World Health Organization. Data from: live life: preventing suicide. (2018). Available at: https://iris.who.int/handle/10665/325650 (Accessed September 3, 2018).

[ref65] World Health Organization. Data from: suicide in the world: global health estimates. (2019). Available at: https://iris.who.int/handle/10665/326948 (Accessed June 16, 2021).

[ref66] Xie N. Qin Y. Wang T. Zeng Y. Deng X. Guan L. (2020). Prevalence of depressive symptoms among nurses in China: a systematic review and meta-analysis. PLoS One 15:e235448:e0235448. doi: 10.1371/journal.pone.0235448, PMID: 32634150 PMC7340293

[ref67] Xu X. Wang W. Chen J. Ai M. Shi L. Wang L.. (2021). Suicidal and self-harm ideation among Chinese hospital staff during the COVID-19 pandemic: prevalence and correlates. Psychiatry Res. 296:113654. doi: 10.1016/j.psychres.2020.113654, PMID: 33360965 PMC7836678

[ref68] Zhang Y. Wu C. Ma J. Liu F. Shen C. Sun J.. (2024). Relationship between depression and burnout among nurses in intensive care units at the late stage of COVID-19: a network analysis. BMC Nurs. 23:224. doi: 10.1186/s12912-024-01867-3, PMID: 38561758 PMC10983623

[ref69] Zhou M. Yao L. Xie L. Wang J. (2004). The mental health situation and its influencing factors in general hospital nurses. Zhonghua Lao Dong Wei Sheng Zhi Ye Bing Za Zhi 22, 435–438. doi: 10.3760/cma.j.issn.1001-9391.2004.06.011, PMID: 15748478

